# *Pristionchus trametes* n. sp. (Diplogastridae) isolated from the mushroom *Trametes orientalis* in Kyoto, Japan

**DOI:** 10.21307/jofnem-2021-060

**Published:** 2021-07-19

**Authors:** Natsumi Kanzaki, Keiko Hamaguchi

**Affiliations:** 1 Kanzsai Research Center, Forestry and Forest Products Research Institute, 68 Nagaikyutaroh, Momoyama, Fushimi, Kyoto, 612-0855, Japan

**Keywords:** Molecular, Morphology, Morphometrics, New Species, Phylogeny, Taxonomy

## Abstract

A new species of *Pristionchus* was isolated from fruiting bodies of the wood-decaying fungus *Trametes orientalis* collected from Kyoto, Japan. Attempts to culture it using bacteria, yeast, and freeze-killed wax moth larvae as food or substrate failed. The eurystomatous form of the species was not found in the collected material, and the species is typologically characterized by: its ‘small’ stoma with thin, membrane-like cheilostomatal plates, a small triangular right subventral tooth, thorn-like dorsal tooth, and small left subventral denticles; a short, blunt male tail spike; and a short, conical female tail. Although the posterior probability support was not high (66%), phylogenetic analysis of both small and large ribosomal RNA gene subunits suggests that the species is closely related to *P. elegans* and *P*. *bucculentus*. The new species can be distinguished from those two by its diagnostic characters comprising the stomatal morphology and male and female tail characters.

The genus *Pristionchus* (Kreis, 1932) consists of approximately 60 nominal species (Herrmann et al., 2015; Kanzaki et al., 2021; Sudhaus and Fürst von Lieven, 2003), and the flagship species of the genus, *P*. *pacificus* Sommer, Carta, kim & Sternburg, 1996, has become a research model organism in many fields, especially in developmental biology due to its environment-dependent stomatal dimorphism, i.e., phenotypic plasticity (e.g., Lightfoot et al., 2019; Ragsdale et al., 2013; Sommer, 2009, 2015). The species in the genus are divergent, and the genus can be used as a model system not only in laboratory studies but also in field ecology, evolutionary biology, and population genetics (e.g., Cinkornpumin et al., 2014; Herrmann et al., 2010; Morgan et al., 2014; Renahan et al., 2021; Rödelsperger et al., 2018).

Most *Pristionchus* species are phoretic and necromenic associates of insects and other invertebrates, and are culturable using *Escherichia coli* strain OP50, a common food source of bacteriophagous nematodes (e.g., Ragsdale et al., 2015). However, one subgeneric clade of *Pristionchus* species associated with figs associates contains several species that are not currently culturable; these species exhibit highly divergent stomatal polymorphism, have specific associations with fig wasps, and inhabit fresh figs (Susoy et al., 2016). The clade may represent the phenotypic and genetic plasticity of the genus.

Despite dense taxon sampling of the genus (e.g., Herrmann et al., 2006; Kanzaki et al., 2021; Mayer et al., 2007), the species diversity of the genus is still far from saturated (Kanzaki et al., 2021; Rödelsperger et al., 2018), and further isolation of various species is necessary to improve the model system.This study describes a species of *Pristionchus* recovered from fruiting bodies (mushrooms) of the wood-decaying fungus *Trametes orientalis* (Yasuda) based on its typological characters and ribosomal RNA sequences, which were used as species-specific molecular barcodes.

## Materials and methods

### Nematode isolation and culturing attempts

No special permit was required for the material collection at the collection site, and no endangered or protected species was involved in the present study.

Several fruiting bodies of *T*. *orientalis* on a dead log were collected from Uji, Kyoto, Japan in July 2020. The wood substrate could not be identified because the bark was lost and the wood was partially decomposed. The fruiting bodies were enclosed in a plastic bag, transported to the laboratory, and examined for nematodes and insects.

Nematodes were collected from the inner surface of the plastic bag after removing the fruiting bodies by washing the bag with distilled water. The fruiting bodies were placed on distilled water agar (2.0% agar) to avoid dehydration and kept in the laboratory at room temperature (20-25°C).

The collected nematodes were briefly observed under light microscopy (Eclipse 80i, Nikon) in differential interference contrast mode to identify genera, and several individuals were transferred to *E*. *coli* OP50 growing on nematode growth medium (NGM). Ten gravid females were transferred to the medium in triplicate, and culture was attempted. In addition, five individual males and females were briefly observed to confirm their typological conspecificity; they were then picked up using a stainless steel insect pin (Insect pin #00, Shiga Kontyu) and transferred to nematode digestion buffer (NDB; Kikuchi et al., 2009; Tanaka et al., 2012) separately to obtain molecular profiles. The remaining nematodes were killed via heating at 55°C for 1 min and fixed in TAF (triethanolamine: formalin: distilled water = 2:7:91) as morphological material.

Because the first culture attempts were unsuccessful, nematodes were collected from the water agar under the fruiting bodies, and we attempted to culture them again using *E*. *coli* OP50 (the above procedure), yeast, freeze-killed *Galleria mellonella* (L.) larvae, and *T*. *orientalis* fruiting bodies.

Entomophilic yeast isolated from a carpenter bee (*Xylocopa appendiculata circumvolans* Smith) were streaked onto 1.0% malt extract agar (MEA: 1.0% malt extract, 2.0% agar), which was then inoculated with 10 gravid female nematodes. For culture with larvae, freeze-killed *G*. *mellonella* larvae were cut in half, placed on 2.0% water agar, and inoculated with nematodes. Next, the *T*. *orientalis* fruiting bodies from which the nematodes were originally isolated were cut into ca. 1.5 × 1.5 cm^2^ pieces and placed on 2.0% water agar; the presence of nematodes was confirmed under a dissecting microscope (S8 Apo; Leica, Wetzlar, Germany). The culture media were kept in the laboratory and observed irregularly for 2 weeks. These attempts were repeated three to five times for each food source tested.

### Morphological observation and micrography

Fixed nematodes were processed with glycerin using Seinhorst’s method with some modifications (Minagawa and Mizukubo, 1994) and mounted in glycerin according to the methods of de Maeseneer and d’Herde (as described in Hooper, 1986). Mounted specimens were used for morphological observations and morphometric analyses, and kept as type specimens. All micrographs were obtained using a digital camera system (MC170 HD; Leica), and morphological drawings were made using a drawing tube connected to the microscope. To prepare the figures, the micrographs and drawings were edited using Photoshop 2019 (Adobe).

### Molecular profiles and phylogeny

The nematodes transferred to NDB were digested at 55°C for 30 min, and the lysates served as template DNA for PCR. To confirm species identity, ca. 0.7 kb of the D2-D3 expansion segments of the large subunit of ribosomal RNA (D2-D3 LSU) was analyzed for all DNA samples following the method in Ye et al. (2007). We then sequenced a 4.2-kb segment of the ribosomal DNA region, including the near-full length sequence of the small subunit (SSU), internal transcribed spacer (ITS) region (ITS1, 5.8S rRNA, and ITS2), and D1 to D4 expansion segments of the large subunit (D1-D4 LSU), as well as partial (ca. 0.7 kb) segments of mitochondrial cytochrome oxidase subunit I (mtCOI), following the methods of Ekino et al. (2017), Kanzaki and Futai (2002), and Kanzaki et al. (2019, 2021). The sequences were deposited in GenBank (https://www.ncbi.nlm.nih.gov/genbank/) under accession numbers LC633357 (rDNA) and LC633358 (mtCOI).

To determine the status of this previously unknown nematode within the genus, a Bayesian phylogenetic analysis was conducted based on concatenated SSU and D1-D4 LSU sequences. First, the compared sequences were aligned using the program MAFFT (Katoh et al., 2002; Kuraku et al., 2013; available at https://mafft.cbrc.jp/alignment/server/index.html) with the default settings. Base-substitution models for each gene were determined using the Akaike information criterion in MEGA X (Kumar et al., 2018). Bayesian analysis was performed using MrBayes 3.2 (Huelsenbeck and Ronquist, 2001; Ronquist et al., 2012): four chains were run for 4 × 10^6^ generations, and the Markov chains were sampled at 100-generation intervals (Larget and Simon, 1999). After performing two independent runs, confirming convergence of the runs, and discarding the first 2 × 10^6^ generations as ‘burn in’, the remaining topologies were used to generate a 50% majority-rule consensus tree. The sequences compared in the analysis are listed in Table 1.

**Table 1.** GenBank accession numbers for the sequences used for the phylogenetic analysis.

Species	SSU	LSU
*Parapristionchus giblindavisi*	JX163981	JX163972
*Pristionchus aerivorus*	FJ040440	KT188862
*Pristionchus americanus*	FJ040445	KT188867
*Pristionchus arcanus*	KT188848	KT188878
*Pristionchus atlanticus*	KT188839	KT188869
*Pristionchus boliviae*	KT188838	KT188868
*Pristionchus borbonicus*	KT188856	KT188885
*Pristionchus brevicauda*	KT188841	KT188871
*Pristionchus bucculentus*	KT188860	KT188889
*Pristionchus bulgaricus*	KT188845	KT188875
*Pristionchus clavus*	KT188842	KT188872
*Pristionchus degawai*	MH114984	–
*Pristionchus elegans*	KJ877238	KJ877274
*Pristionchus entomophagus*	FJ040441	KT188873
*Pristionchus exspectatus*	KT188849	KT188879
*Pristionchus fissidentatus*	KT188855	KJ877273
*Pristionchus fukushimae*	KT188852	KT188882
*Pristionchus hongkongensis*	MH114985	–
*Pristionchus hoplostomus*	KT188853	KT188883
*Pristionchus japonicus*	KT188850	KT188880
*Pristionchus laevicollis*	MH114986	–
*Pristionchus lheritieri* SB245	KT188846	KT188876
*Pristionchus lucani*	KT188844	KT188874
*Pristionchus marianneae*	FJ040442	KT188866
*Pristionchus maupasi*	FJ040443	KT188863
*Pristionchus maxplancki*	KT188851	KT188881
*Pristionchus mayeri*	KT188835	KT188865
*Pristionchus neolucani*	MH114987	–
*Pristionchus occultus*	KX113518	–
*Pristionchus pacificus* PS312	AF083010	EU195982
*Pristionchus pauli*	FJ040446	KT188870
*Pristionchus paulseni*	MH114982	–
*Pristionchus pseudaerivus*	FJ040447	KT188864
*Pristionchus quartusdecimus*	KT188847	KT188877
*Pristionchus racemosae*	KT188859	KT188888
*Pristionchus riukiariae*	MH114988	–
*Pristionchus sycomori*	KT188857	KT188886
*Pristionchus triformis*	KT188854	KT188884
*Pristionchus uniformis*	KJ877236	KJ877272
*Pristionchus yamagatae*	MH114983	–
*Pristionchus* sp. 2 CW-2016	KX113519	–
*Pristionchus* sp. 3 CW-2016	KX113517	–
*Pristionchus* sp. 35 VS-2015	KT188858	KT188887
*Pristionchus* sp. 38 VS-2015	KT188861	KT188890
*Pristionchus trametes* n. sp.	LC633357	

## Description

**Figure 1:**
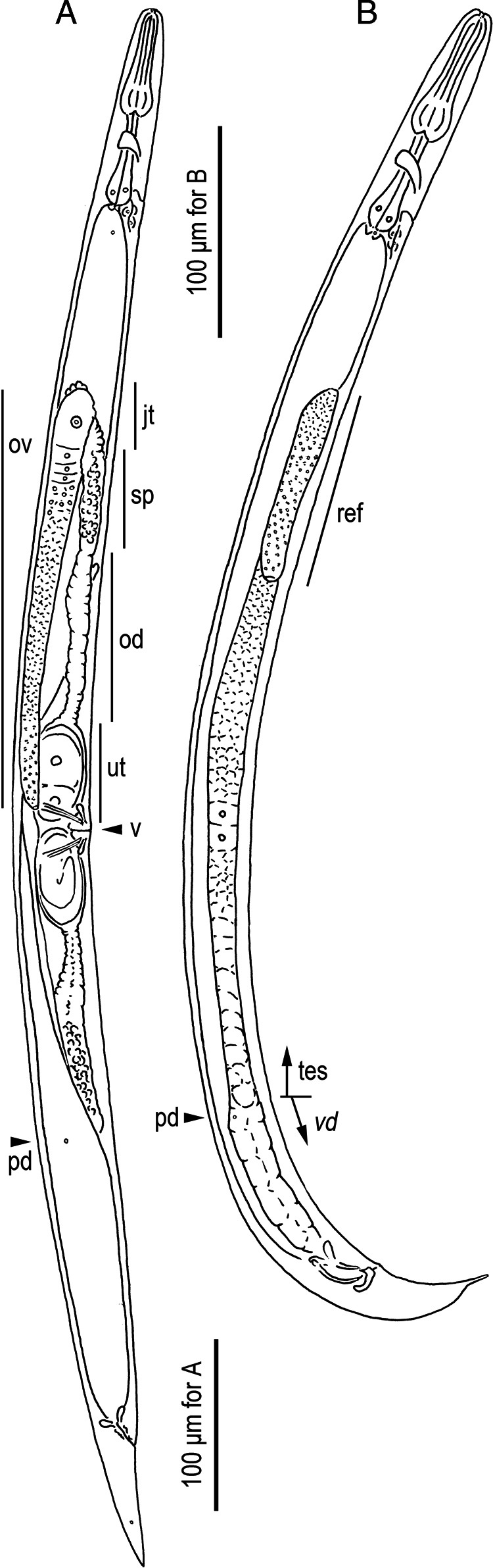
*Pristionchus trametes* n. sp. adults in the right lateral view. A: Female (ov, ovary; jt, junction tissue; sp, spermatheca; od, oviduct; ut, uterus; v, vulva; pd, postdeirid). B: Male (ref, reflexed part of testis; tes, testis; *vd*, *vas deferens*).

**Figure 2:**
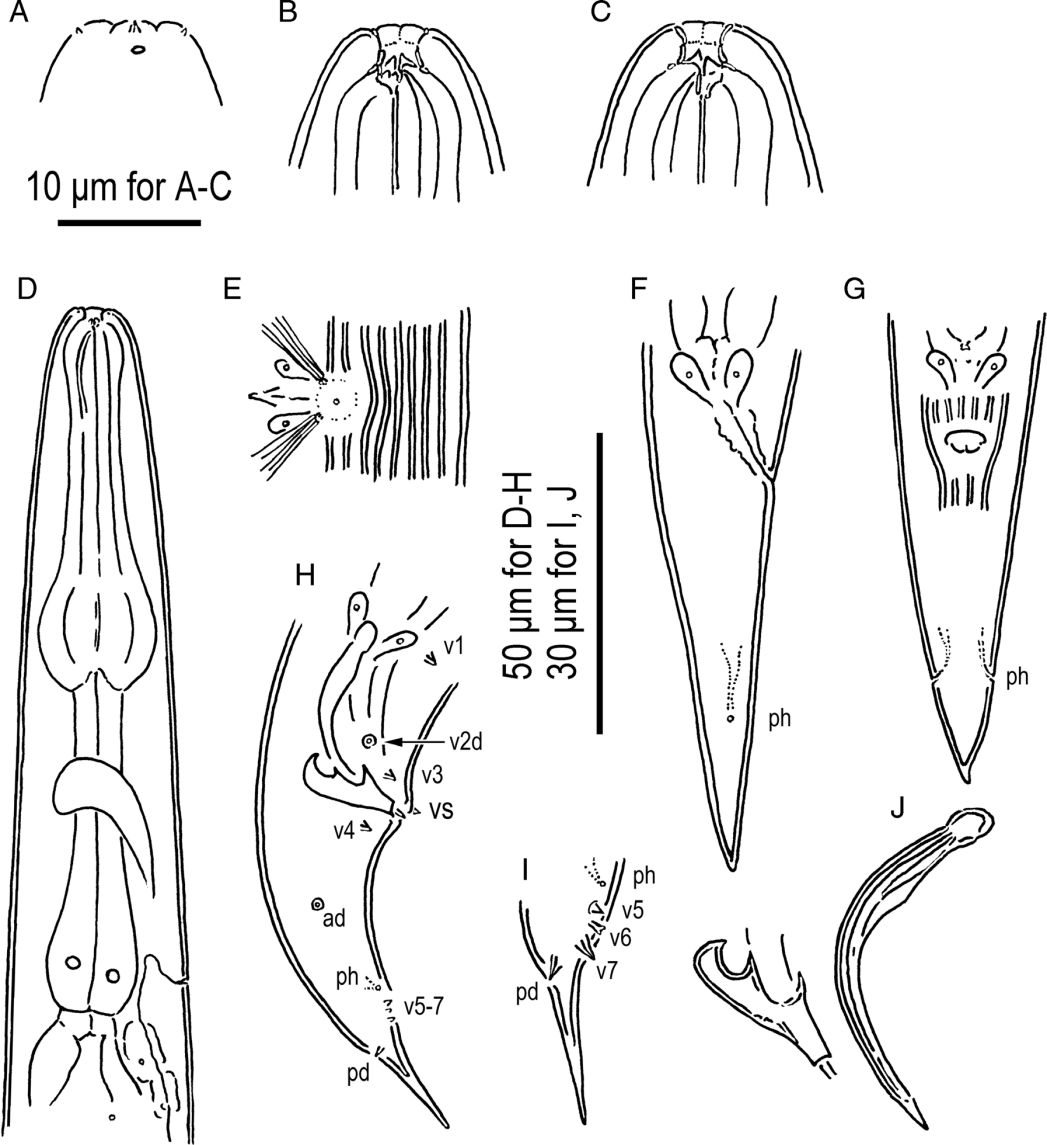
*Pristionchus trametes* n. sp. adults. A: Surface of the lip region. B, C: Stomatal region. D: Anterior part of an adult. E: Vulval region. F, G: Female tail (ph, phasmid). H: Male tail (v + number, ad, genital papillae where “d” indicates laterally oriented papillae; ph, phasmid). I: Male tail tip (labels are same as H). J: Spicule and gubernaculum. A and B, left lateral view; E and G, ventral view; the others, right lateral view.

**Figure 3:**
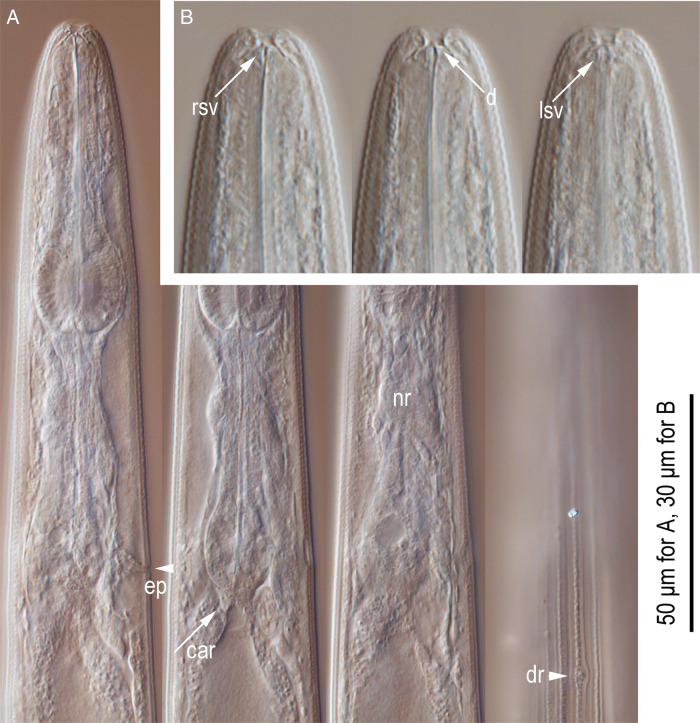
*Pristionchus trametes* n. sp. adults.A: Right lateral view of the stoma-pharyngeal region in four different focal planes (ep, secretory-excretory pore; car, cardia; nr, nerve ring; dr, deirid). B: Left lateral view of the stomatal region in three different focal planes (rsv, right subventral tooth; d, dorsal tooth; lsv, left subventral denticles).

**Figure 4:**
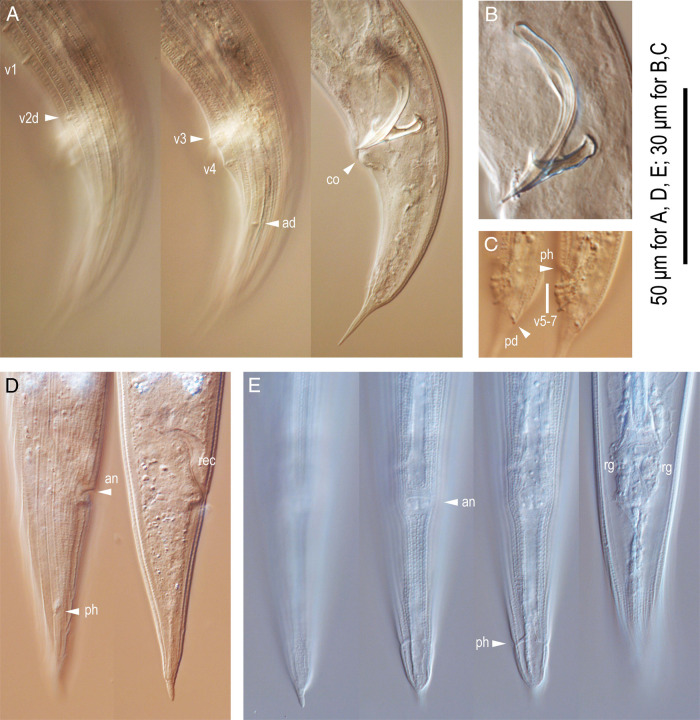
*Pristionchus trametes* n. sp. adults. A: Left lateral view of the entire male tail (v + number, ad, genital papillae where “d” indicates laterally oriented papillae; co, cloacal opening). B: Spicule and gubernaculum in left lateral view. C: Left lateral view of the distal part of the male tail in two different focal planes (v + number, pd, genital papillae; ph, phasmid). D: Right lateral view of the female tail in two different focal planes (an, anus; ph, phasmid; rec, rectum). E: Ventral view of the female tail in four different focal planes (an, anus; ph, phasmid; rg, rectal glands).

*Pristionchus trametes* * n. sp. (Figs. 1-4 and Table 1).

### Measurements

Summarized in Table 2.

**Table 2.** Morphometric values for *Pristioinchus trametes* n. sp.

	Male		Female
	Holotype	Paratypes	Paratypes
*N*	–	9	10
*L*	712	696 ± 58 (615–785)	819 ± 41 (746–893)
*a*	24.1	23.9 ± 1.7 (20.9–26.1)	19.9 ± 0.7 (18.4–20.9)
*b*	6.3	6.5 ± 0.5 (5.9–7.6)	7.2 ± 0.4 (6.7–7.6)
*c*	12.4	11.9 ± 0.6 (11.1–13.3)	12.3 ± 1.2 (10.2–13.9)
*c′*	2.4	2.4 ± 0.1 (2.2–2.6)	3.6 ± 0.5 (2.8–4.4)
T or V	78.0	84.5 ± 3.3 (77.6–88.9)	51.6 ± 0.8 (50.4–52.8)
Anterior pharynx	59	55 ± 2.8 (49–59)	57 ± 3.0 (54–64)
Posterior pharynx	49	48 ± 2.2 (43–52)	51 ± 3.1 (45–55)
Anterior/posterior pharynx ratio	1.1	1.1 ± 0.1 (1.0–1.2)	1.1 ± 0.1 (1.0–1.2)
Nerve ring from anterior end	79	75 ± 3.9 (68–81)	77 ± 4.0 (72–85)
Secretory-excretory pore from anterior end	102	99 ± 5.0 (93–110)	104 ± 5.0 (96–112)
Deirid from anterior end	117	116 ± 4.9 (110–126)	127 ± 5.0 (117–134)
Median bulb diam.	17.6	16.9 ± 0.7 (15.8–18.0)	19.0 ± 0.7 (18.0–19.8)
Basal bulb diam.	14.3	12.9 ± 1.0 (11.0–14.4)	14.3 ± 0.7 (13.3–15.1)
Maximum body diam.	30	29 ± 3.2 (25–36)	41 ± 2.4 (37–43)
Cloacal or anal body diam.	24	24 ± 1.6 (22–27)	19 ± 1.1 (14–22)
Tail length	58	59 ± 3.9 (52–65)	67 ± 7.1 (60–79)
Gonad length	553	588 ± 50 (519–668)	–
*Vas deferens* length	102	110 ± 14 (95–136)	–
Ratio of *vas deferens* to total gonad (%)	18	19 ± 1.3 (17–21)	
Spicule (arc)	40	39 ± 2.1 (36–42)	–
Spicule (chord)	33	31 ± 1.7 (29–33)	–
Gubernaculum (chord)	16.8	17.6 ± 1.4 (15.8–19.9)	–
Tail spike length	13.8	12.9 ± 2.1 (9.2–16.8)	–
Ratio (%) of tail spike to total tail	24	22 ± 2.9 (16–26)	–
Anterior ovary	–	–	255 ± 17 (229–278)
Posterior ovary	–	–	286 ± 54 (214–363)
Vulval body diam.	–	–	41 ± 2.1 (37–43)
Anus-phasmid	–	–	38 ± 4.1 (34–48)
Relative position of phasmid to total tail (%)	–	–	57 ± 5.6 (48–66)

### Adult

Body cylindrical, stout. Cuticle thick, with fine annulation and clear longitudinal striations. Lateral field consisting of two lines, only weakly distinguishable from body striation with the presence of deirid. Head without apparent lips, and with six short and papilliform labial sensillae; four small, papilliform cephalic papillae present in males, as typical for diplogastrid nematodes. Amphidial apertures located on the lateral sector, slightly dorsally shifted, at level of margin of cheilo- and gymnostom. However, labial and cephalic papillae and amphidial aperture were not observed clearly in the most individuals examined. Stomatal dimorphism often found in the genus not observed, and all individual have ‘small’ (short and narrow) stoma, hypothesized to be stenostomatous form. Cheilostom consisting of six thin, membrane-like per- and interradial plates. Incision between plates not easily distinguished by LM observation.

### Male

Ventrally arcuate, strongly ventrally curved at tail region when killed by heat. Testis single, ventrally located, anterior part reflexed to right side; spermatogonia arranged in three to five rows in reflexed part, then well-developed spermatocytes arranged as three to four rows in anterior half of the main branch, then mature ameboid spermatocytes arranged in single to two row(s) in proximal part of gonad. *Vas deferens* not clearly separated from other parts of gonad, formed by relatively large cells. Three (two subventral and one dorsal) cloacal gland cells observed at distal end of *vas deferens* and intestine. Spicules paired, separate; spicules smoothly curved in ventral view, adjacent to each other for distal third of their length, each smoothly tapering to pointed distal end. Spicule in lateral view smoothly ventrally arcuate, giving spicule about 100° curvature, oval manubrium present at anterior end, lamina/calomus complex (blade) clearly expanded slightly posterior to manubrium (*ca*. 1/5 of blade length from anterior), then smoothly tapering to pointed distal end. Gubernaculum conspicuous, about one-third of spicule length, broad anteriorly such that dorsal wall is slightly recurved with dorsal and ventral walls separate at 50 to 60° angle at posterior end; dorsal side of gubernaculum possessing single, membranous, anteriorly directed process and lateral pair of more sclerotized, anteriorly and obliquely ventrally directed processes. In lateral view, anterior half of gubernaculum with two serial curves separated by anteriorly and obliquely ventrally directed process, with anterior terminal curvature highly concave and almost closed, and with deep posterior curvature being one-third of gubernaculum length; posterior half forming tube-like process enveloping spicules. Cloacal opening (CO) forming simple slit. One small, ventral, single genital papilla (vs) on anterior cloacal lip; nine pairs of genital papillae (v1-v7, ad, pd) and a pair of phasmids (ph) present, with an arrangement <v1, (v2d, v3/v4), ad, (ph, V5-7, pd)> in nomenclature of Sudhaus and Fürst von Lieven, 2003), where v1 is located at *ca* 1.5 cloacal body diam. (CBD) anterior to CO; v2d to v4 are close to each other, gathering within less than half CBD; ad at *ca*. 1 CBD posterior to CO; and ph, v5-7 and pd close to each other around immediately anterior to the root of tail spike. Anterior five pairs of papillae (v1-4 and ad) almost equal in size, rather large and conspicuous, v7 and pd papillae obviously smaller than anterior five pairs, v5 and v6, sometimes difficult to observe with light microscope. Anterior two pairs of the ventral triplet papillae (v5 and v6) papilliform and borne from socket-like base, v7 simple or typical thorn-like in shape. Tip of v6 papillae split into two small papilla-like projections. Tail conical, with short, *ca*. 0.6 CBD in length, spike with bluntly pointed tip. Bursa or bursal flap absent.

### Female

Relaxed or slightly ventrally arcuate when killed by heat. Gonad didelphic, amphidelphic; each gonadal system arranged from vulva/vagina as uterus, oviduct, and ovary. Anterior and posterior gonads are basically same in their structure, and only anterior gonad is described in detail here. Anterior gonad right of intestine, with uterus and oviduct extending ventrally and anteriorly on right of intestine and with a totally reflexed (= antidromous reflexion) ovary extending dorsally. Oocytes in ovary mostly arranged in three to four rows in distal two-thirds to three-fourth of ovary and in double or single row in rest of ovary, distal tip ovary reaching vulva to oviduct of opposite gonad branch depending on the developmental condition. Anterior end of oviduct (= junction tissue between ovary and oviduct) consists of rounded cells. Spermatheca not clearly distinct, but anterior part of oviduct immediately posterior to the junction consists of rounded cells works as spermatheca. Eggs in single to multiple-cell stage or even further developed at posterior part of oviduct (= uterus). *Receptaculum seminis* not observed, i.e., the organ is not independent, and a part of oviduct/uterus works as the organ. Vaginal glands present but obscure. Vagina perpendicular to body surface, surrounded by sclerotized tissue; vulva slightly protuberant in lateral view, pore-like in ventral view. Rectum about one anal body diameter long, intestine/rectum junction surrounded by well-developed sphincter muscle; three anal glands (two subventral and one dorsal) present but not obvious. Anus in form of dome-shaped slit, posterior anal lip slightly protuberant. Phasmid about two anal body diam. posterior to anus, or middle to 1/2-2/3 tail length posterior to anus. Tail short, conical with bluntly pointed or narrowly rounded terminus.

### Molecular profile and phylogeny

**Figure 5:**
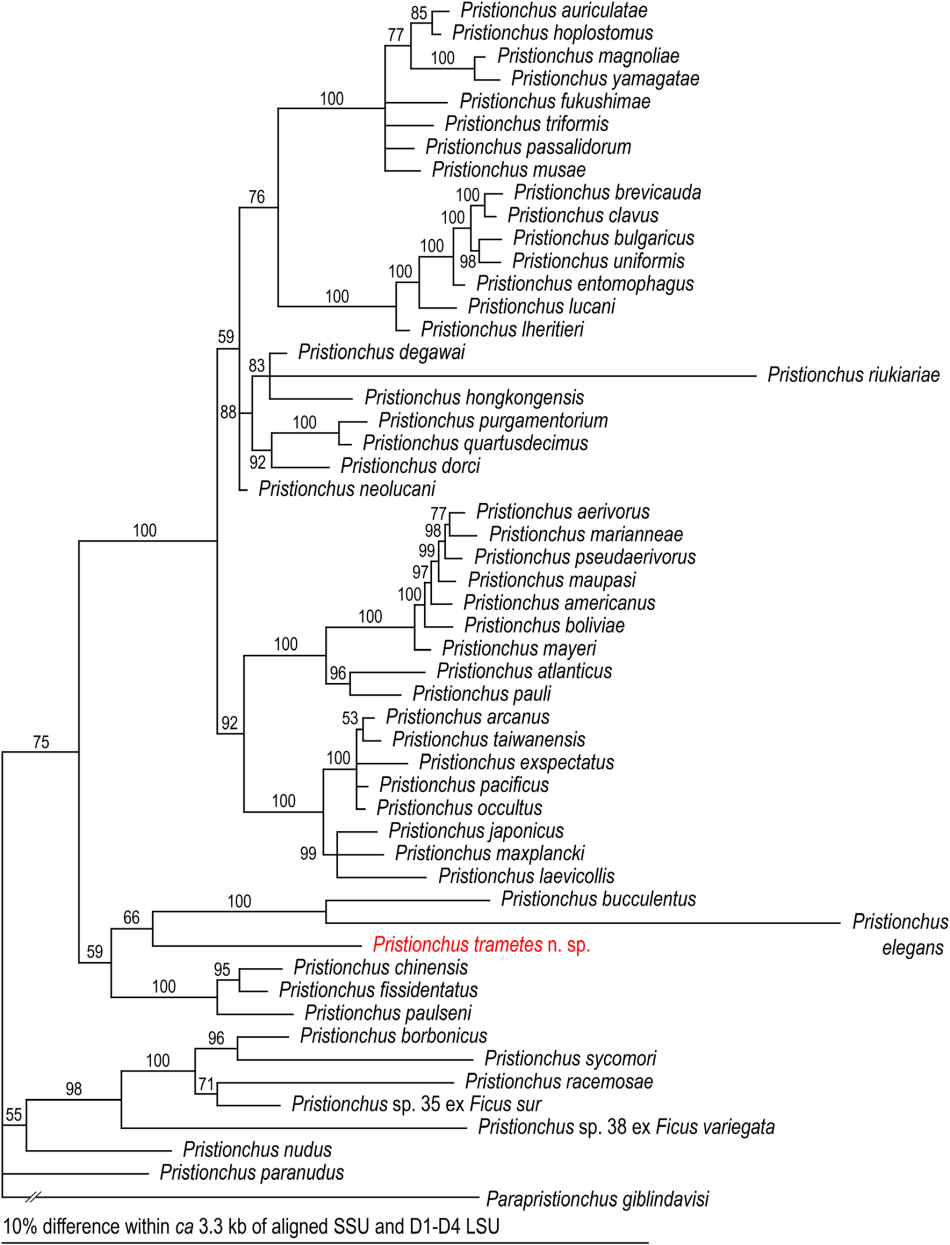
Phylogenetic relationships of *Pristionchus* spp. A Bayesian tree was inferred from concatenated SSU and D1-D4 LSU sequences under the GTR + G + I model. The following analytical parameters were used: SSU, freqA = 0.25, freqC = 0.21, freqG = 0.26, freqT = 0.28, R(a) = 0.77, R(b) = 2.52, R(c) = 2.09, R(d) = 0.83, R(e) = 3.32, R(f) = 1.00, Pinva = 0.46, Shape = 0.48; D1-D4 LSU, freqA = 0.23, freqC = 0.22, freqG = 0.31, freqT = 0.25, R(a) = 0.57, R(b) = 2.43, R(c) = 0.94, R(d) = 0.68, R(e) = 4.39, R(f) = 1.00, Pinva = 0.39, Shape = 0.49. Posterior probability values exceeding 50% are shown for each clade.

Although the posterior probability support was low (66%), *P*. *trametes* n. sp. formed a clade with the two species of the *elegans* group: *P*. *elegans* Kanzaki, Ragsdale, Herrmann & Sommer, 2012 and *P*. *bucculentus* Kanzaki, Ragsdale, Herrmann, Röseler & Sommer, 2013 (Fig. 5).

### Type locality and habitat

The type material was isolated in July 2020 from fruiting bodies of *T. orientalis* collected at Uji, Kyoto, Japan (34°52′31″N, 135°48′03″E, 109 m a.s.l.).

### Type designation and deposition

A holotype male (United States Department of Agriculture Nematode Collection [USDANC] accession number T-756t), four paratype males (T-7500p-7503p), and five paratype females (T-7504p-7508p) were deposited in the USDANC (Beltsville, MD, USA), and vouchers of five paratype males (accession number *Pristionchus trametes* M-01-05) and five paratype females (accession number *Pristionchus trametes* F-01-05) were deposited at the Forest Pathology Laboratory, Forestry and Forest Products Research Center (FFPRI), Tsukuba, Japan. In addition to the type material, several samples fixed in TAF or processed in dehydrated glycerin were deposited at the Kansai Research Center of FFPRI.

### Differential diagnosis

*Pristionchus trametes* n. sp. is characterized by its short, narrow stoma with thin membrane-like cheilostomatal plates, small inverted ‘V’-shaped dorsal tooth, triangular right subventral tooth with a blunt tip, and left subventral plate with three blunt denticules; male genital papillae with a v1, (v2d, v3/v4), ad, (ph, V5-7, pd) arrangement and short blunt male tail spike; and short conical female tail with a bluntly pointed or narrowly rounded terminus.

Based on its membrane-like cheilostomatal plates, the new species is close in form to two *elegans* group species, *P*. *elegans* and *P*. *bucculentus*; these three species share this unique morphological character as well as a similar arrangement of genital papillae, i.e., the second pair of genital papillae is oriented laterally (v2d), and the second to fourth pairs (v2d, v3, and v4) are close to each other (Kanzaki et al., 2012, 2013). Although phylogenetic support for a grouping of all three was low, these three species formed a subclade within the genus in our phylogenetic analysis (Fig. 5).

Distinguishing *P*. *trametes* n. sp. from *P*. *elegans* are stomatal characters, although only a single (stenostomatous) form is known for the two species. Diagnostic characters of *P. trametes* n. sp., with respect to *P. elegans*, are the absence vs. presence of anterior serratae of the gymnostomatal tube and a triangular vs. flattened right subventral tooth. In terms of the relative length of anterior and posterior pharynx, the anterior pharynx of *P*. *trametes* n. sp. is slightly (1.1 times) vs. obviously (1.5 times) longer than the posterior pharynx. The position of male v1 paired papillae in *P*. *trametes* n. sp. is 1.5 vs. less than 1 CBD anterior to CO, the male tail tip has a short spike vs. a long and filiform appendage, and the female tail is short and conical vs. long and filiform (Kanzaki et al., 2012). The new species is also distinguished from *P*. *bucculentus* by its stomatal shape, which shows only the stenostomatous vs. only the eurystomatous form, relative length of the anterior and posterior pharynx, with its anterior pharynx slightly (1.1 times) vs. obviously (1.5 times) longer than the posterior pharynx, the position of male v1 paired papillae (1.5 vs. ca. 1 CBD anterior to CO), a male tail tip with a short spike vs. a long and filiform appendage, and a short and conical *vs*. long and filiform female tail (Kanzaki et al., 2013).

### Additional remarks on nematode isolation and culture attempts

In addition to the specimens initially collected from the plastic bag including the *T. orientalis* specimens, several aphelenochoidids, rhabditids, and plectids were recognized in the 2.0% water agar where the small pieces of *T*. *orientalis* were placed. No predation behavior of *P*. *trametes* n. sp. was seen during occasional observations.

Regardless of the methodology, none of the culture attempts was successful, i.e., no oviposition or propagation of *P*. *trametes* n. sp. was observed in the cultures. Further, several unidentified dipteran larvae and adult mushroom beetles (Coleoptera: Ciidae) infected the fruiting bodies placed on the 2.0% water agar, and some died on the medium. Although several mushroom beetles harbored other entomophilic rhabditids, *P*. *trametes* n. sp. was not observed on these dead bodies; therefore, an insect association of *P*. *trametes* n. sp. was not confirmed.

## Discussion

This study describes a new species of *Pristionchus*. Because the isolates were not cultured successfully, several morphological characters were not observed sufficiently. For example, the labial and cephalic sensilla were not observed in many individuals. This could be because of the material condition, although males in a recently described species *P*. *nudus* Kanzaki, Herrmann, Weiler, Röseler, Theska, Berger, Rödelsperger & Sommer, 2021 had lost cephalic sensilla (Kanzaki et al., 2021). Similar degeneration might have occurred in *P*. *trametes* n. sp. Further detailed morphological observations of live specimens might elucidate these fine characters.

*Pristionchus trametes* n. sp. has several characteristic morphological traits that are uncommon in the genus. For example, the stomatal structure of the new species is unique. Stomatal dimorphism was not observed in this study; because of its “small” size, the species is tentatively considered monomorphic with a stenostomatous form. However, considering its teeth shape, the form seems intermediate between two forms, i.e., the anterior tip of the dorsal tooth curved slightly anteriorly, and the right subventral tooth is relatively large compared with the stoma size. In addition, the tail morphology (short, spiked in males and short, conical in females) is not found in *Pristionchus* spp. except for *P*. *chinensis* Kanzaki, Herrmann, Weiler, Röseler, Theska, Berger, Rödelsperger & Sommer, 2021, which belongs to the group that is sister to thre rest of the genus, excluding the clade of fig associates (Kanzaki et al., 2021).

In some respects, tail and stomatal morphology is similar to that of other diplogastrid genera. For example, the stomatal form, comprising a buccal cavity with two teeth, is similar to that of several *Parasitodiplogaster* Poinar, 1979 species in which the relatively narrow stoma has two teeth (e.g., Giblin-Davis et al., 2006; Wöhr et al., 2014), and several species of *Allodiplogaster* Paramonov and Sobolev in Skrjabin et al. (1954) have a short, spiked male tail and conical female tail (e.g., Kanzaki et al., 2015). Because *Allodiplogaster* is clearly distant from *Pristionchus*, representing a basal split within the family with respect to the latter genus (Susoy et al., 2015), these traits are likely to be independent conversions. The characteristic stomatal structure of the new species is likely related to the difficulty in culturing it. Comparative studies of their biological characters may give new insight into their functional morphology and feeding (nutritional) preferences.

Biologically, the new species was found in the fruiting bodies of a wood-decaying fungus, and its insect associations are unclear. *Pristionchus bucculentus*, a close relative of the new species, has been isolated from mushroom beetles in Japan, i.e., *Episcapha gorhami* Lewis from Hokkaido (Kanzaki et al., 2013) and *Encaustes praenobilis* Lewis from Aomori (Kanzaki et al., 2014). These two species may share similar habitats, and an examination of mushroom substrates and related insects may reveal further diversity of mushroom-associated *Pristionchus* species.

## Footnotes

* The species epithet was derived from the generic name (*Trametes*) of the fruiting body substrate from which the new species was recovered.

## References

[ref001] Cinkornpumin, J. K. , Wisidagama, D. R. , Rapoport, V. , Go, J. L. , Dieterich, C. , Wang, X. , Sommer, R. J. and Hong, R. L. 2014. A host beetle pheromone regulates development and behavior in the nematode *Pristionchus pacificus* . eLife 3:e03229, doi: 10.7554/eLife.03229. PMC4270288 25317948

[ref002] Ekino, T. , Yoshiga, T. , Takeuchi-Kaneko, Y. and Kanzaki, N. 2017. Transmission electron microscopic observation of body cuticle structures of phoretic and parasitic stages of Parasitaphelenchinae nematodes. PLoS ONE 12:e0179465, doi: 10.1371/journal.pone.0179465. 28622353 PMC5473575

[ref003] Giblin-Davis, R. M. , Ye, W. , Kanzaki, N. , Williams, D. , Morris, K. and Thomas, W. K. 2006. Stomatal ultrastructure, molecular phylogeny, and description of *Parasitodiplogaster laevigata* n. sp. (Nematoda: Diplogastridae), a parasite of fig wasps from *Ficus laevigata* from Florida. Journal of Nematology 38:137–149. 19259439 PMC2586439

[ref004] Herrmann, M. , Mayer, W. E. and Sommer, R. J. 2006. Sex, bugs and Haldane’s rule: the nematode genus *Pristionchus* in the United States. Frontiers in Zoology 3:14, doi: 10.1186/1742-9994-3-14. 16968539 PMC1578557

[ref005] Herrmann, M. , Kienle, S. , Rochat, J. , Mayer, W. and Sommer, R. J. 2010. Haplotype diversity of the nematode *Pristionchus pacificus* on Réunion in the Indian Ocean suggests multiple independent invasions. Biological Journal of the Linnean Society 100:170–179, doi: 10.1111/j.1095-8312.2010.01410.x.

[ref006] Herrmann, M. , Neuner, J. , Voetsch, M. , Wörner, J. , Kanzaki, N. and Sommer, R. J. 2015. *Pristionchus* Scratchpads – an online platform for taxonomy, systematics and phylogeny. Zootaxa 3949:597–600, doi: 10.11646/zootaxa.3949.4.10. 25947829

[ref007] Hooper, D. J. 1986. “Handling, fixing, staining and mounting nematodes”, In Southey, J. F. (Ed.), Laboratory Methods for Work with Plant and Soil Nematodes Her Majesty’s Stationery Office, London, pp. 59–80.

[ref008] Huelsenbeck, J. P. and Ronquist, F. 2001. MR BAYES: Bayesian inference of phylogenetic trees. Bioinformatics 17:1754–1755, doi: 10.1093/bioinformatics/17.8.754. 11524383

[ref009] Kanzaki, N. and Futai, K. 2002. A PCR primer set for determination of phylogenetic relationships of *Bursaphelenchus* species within *xylophilus* group. Nematology 4:35–41, doi: 10.1163/156854102760082186.

[ref010] Kanzaki, N. , Giblin-Davis, R. M. and Ragsdale, E. J. 2015. *Allodiplogaster josephi* n. sp. and *A. seani* n. sp. (Nematoda: Diplogastridae), associates of soil-dwelling bees in the eastern USA. Nematology 17:831–863, doi: 10.1163/15685411-00002908.

[ref011] Kanzaki, N. , Taki, H. , Masuya, H. and Okabe, K. 2014. Isolation of *Pristionchus bucculentus* from the large mushroom beetle, *Encaustes praenobilis* . Bulletin of Forestry and Forest Products Research Institute 13:27–28.

[ref012] Kanzaki, N. , Ragsdale, E. J. , Herrmann, M. and Sommer, R. J. 2012. Two new species of *Pristionchus* (Rhabditida: Diplogastridae): *P. fissidentatus* n. sp. from Nepal and La Réunion Island and *P. elegans* n. sp. from Japan. Journal of Nematology 44:80–91. 23483847 PMC3593256

[ref013] Kanzaki, N. , Ragsdale, E. J. , Herrmann, M. , Röseler, W. and Sommer, R. J. 2013. *Pristionchus bucculentus* n. sp. (Rhabditida: Diplogastridae) isolated from a shining mushroom beetle (Coleoptera: Scaphidiidae) in Hokkaido, Japan. Journal of Nematology 45:78–86. 23589663 PMC3625135

[ref014] Kanzaki, N. , Masuya, H. , Ichihara, Y. , Maehara, N. , Aikawa, T. , Ekino, T. and Ide, T. 2019. *Bursaphelenchus carpini* n. sp., *B. laciniatae* n. sp. and *B. cryphali okhotskensis* n. subsp. (Nematoda: Aphelenchoididae) isolated from Japan. Nematology 21:361–388, doi: 10.1163/15685411-00003220.

[ref015] Kanzaki, N. , Herrmann, M. , Weiler, C. , Röseler, W. , Theska, T. , Berger, J. , Rödelsperger, C. and Sommer, R. J. 2021. Nine new *Pristionchus* (Nematoda: Diplogastridae) species from China. Zootaxa 4943:1–66, doi: 10.11646/zootaxa.4943.1. 33757041

[ref016] Katoh, K. , Misawa, K. , Kuma, K. and Miyata, T. 2002. MAFFT: a novel method for rapid multiple sequence alignment based on fast Fourier transform. Nucleic Acids Research 30:3059–3066, doi: 10.1093/nar/gkf436. 12136088 PMC135756

[ref017] Kikuchi, T. , Aikawa, T. , Oeda, Y. , Karim, N. and Kanzaki, N. 2009. A rapid and precise diagnostic method for detecting the pinewood nematode *Bursaphelenchus xylophilus* by loop-mediated isothermal amplification (LAMP). Phytopathology 99:1365–1369. 19900002 10.1094/PHYTO-99-12-1365

[ref018] Kreis, H. A. 1932. Beiträge zur Kenntnis pflanzenparasitischer Nematoden. Zeitschrift für Parasitenkunde 5:184–194.

[ref019] Kumar, S. , Stecher, G. , Li, M. , Knyaz, C. and Tamura, K. 2018. MEGA X: molecular evolutionary genetics analysis across computing platforms. Molecular Biology and Evolution 35:1547–1549, doi: 10.1093/molbev/msy096. 29722887 PMC5967553

[ref020] Kuraku, S. , Zmasek, C. M. , Nishimura, O. and Katoh, K. 2013. aLeaves facilitates on-demand exploration of metazoan gene family trees on MAFFT sequence alignment server with enhanced interactivity. Nucleic Acids Research 41:W22–W28, doi: 10.1093/nar/gkt389. 23677614 PMC3692103

[ref021] Larget, B. and Simon, D. L. 1999. Markov chain Monte Carlo algorithms for the Bayesian analysis of phylogenetic trees. Molecular Biology and Evolution 16:750–759, doi: 10.1093/oxfordjournals.molbev.a026160.

[ref022] Lightfoot, J. W. , Wilecki, M. , Rödelsperger, C. , Moreno, E. , Susoy, V. , Witte, H. and Sommer, R. J. 2019. Small peptide–mediated self-recognition prevents cannibalism in predatory nematodes. Science 364:86–89, doi: 10.1126/science.aav9856. 30948551

[ref023] Mayer, W. E. , Herrmann, M. and Sommer, R. J. 2007. Phylogeny of the nematode genus *Pristionchus* and implications for biodiversity, biogeography and the evolution of hermaphroditism. BMC Evolutionary Biology 7:104, doi: 10.1186/1471-2148-7-104. 17605767 PMC1929057

[ref024] Minagawa, N. and Mizukubo, T. 1994. A simplified procedure of transferring nematodes to glycerol for permanent mounts. Japanese Journal of Nematology 24:75, doi: 10.3725/jjn1993.24.2_75.

[ref025] Morgan, K. , McGaughran, A. , Ganeshan, S. , Herrmann, M. and Sommer, R. J. 2014. Landscape and oceanic barriers shape dispersal and population structure in the Island nematode *Pristionchus pacificus* . Biological Journal of the Linnean Society 112:1–15, doi: 10.1111/bij.12255.

[ref026] Poinar, G. O. Jr 1979. *Parasitodiplogaster sycophilon* gen. n., sp. n. (Diplogasteridae: Nematoda), a parasite of *Elisabethiella stuckenbergi* Grandi (Agaonidae: Hymenoptera) in Rhodesia. Proceedings of the Koninklijke Nederlandse Akademie van Wetenschappen, Series C, Biological and Medical Sciences 82:375–381.

[ref027] Ragsdale, E. J. , Kanzaki, N. and Herrmann, M. 2015. “Taxonomy and natural history: the genus *Pristionchus*”, In Sommer, R. J. , Hunt, D. J. and Perry, R. N. (Eds), *Pristionchus Pacificus* – a Nematode Model for Comparative and Evolutionary Biology. Nematology Monographs and Perspectives vol. 11 Brill, Leiden, pp. 77–120.

[ref028] Ragsdale, E. J. , Müller, M. R. , Rödelsperger, C. and Sommer, R. J. 2013. A developmental switch coupled to the evolution of plasticity acts through a sulfatase. Cell 155:922–933, doi: 10.1016/j.cell.2013.09.054. 24209628

[ref029] Renahan, T. , Lo, W. -S. , Werner, M. S. , Rochat, J. , Herrmann, M. and Sommer, R. J. 2021. Nematode biphasic “boom and bust” dynamics are dependent on host bacterial load while linking dauer and mouth-form polyphenisms. Environmental Microbiology 23, doi: 10.1111/1462-2920.15438. 33587771

[ref030] Rödelsperger, C. , Röseler, W. , Prabh, N. , Yoshida, K. , Weiler, C. , Herrmann, M. and Sommer, R. J. 2018. Phylotranscriptomics of *Pristionchus* nematodes reveals parallel gene loss in six hermaphroditic lineages. Current Biology 28:3123–3127, doi: 10.1016/j.cub.2018.07.041. 30245109

[ref031] Ronquist, F. , Teslenko, M. , van der Mark, P. , Ayres, D. L. , Darling, A. , Höhna, S. , Larget, B. , Liu, L. , Suchard, M. A. and Huelsenbeck, J. P. 2012. MrBayes 3.2: efficient Bayesian phylogenetic inference and model choice across a large model space. Systematic Biology 61:539–542, doi: 10.1093/sysbio/sys029. 22357727 PMC3329765

[ref032] Skrjabin, K. I. , Shikobalova, N. P. , Sobolev, A. A. , Paramonov, A. A. and Sudarikov, A. A. 1954. Camellanata, Rhabditata, Tylenchata, Trichocephalata and Dioctophymata and the distribution of parasitic nematodes by hosts. Izdatel’stvo Akademii Nauk SSSR (Moskva) 4:1–927 (in Russian).

[ref033] Sommer, R. J. 2009. The future of evo–devo: model systems and evolutionary theory. Nature Reviews Genetics 10:416–422, doi: 10.1038/nrg2567. 19369972

[ref034] Sommer, R. J. 2015. “Integrative evolutionary biology and mechanistic approaches in comparative biology”, In Sommer, R. J. , Hunt, D. J. and Perry, R. N. (Eds), *Pristionchus Pacificus* – a Nematode Model for Comparative and Evolutionary Biology. Nematology monographs and perspectives vol. 11 Brill, Leiden, pp. 19–41.

[ref035] Sommer, R. J. , Carta, L. K. , Kim, S. -Y. and Sternberg, P. W. 1996. Morphological, genetic and molecular description of *Pristionchus pacificus* sp. n. (Nematoda, Diplogastridae). Fundamental and Applied Nematology 19:511–521.

[ref036] Sudhaus, W. and Fürst von Lieven, A. 2003. Phylogenetic classification of the Diplogastridae (Secernentea, Nematoda). Journal of Nematode Morphology and Systematics 6:43–90.

[ref037] Susoy, V. , Ragsdale, E. J. , Kanzaki, N. and Sommer, R. J. 2015. Rapid diversification associated with a macroevolutionary pulse of developmental plasticity. eLife 4:e05463, doi: 10.7554/eLife.05463. PMC4357287 25650739

[ref038] Susoy, V. , Herrmann, M. , Kanzaki, N. , Kruger, M. , Nguyen, C. N. , Rödelsperger, C. , Röseler, W. , Weiler, C. , Giblin-Davis, R. M. , Ragsdale, E. J. and Sommer, R. J. 2016. Large-scale diversification without genetic isolation in nematode symbionts of figs. Science Advances 2:e1501031, doi: 10.1126/sciadv.1501031. 26824073 PMC4730855

[ref039] Tanaka, R. , Kikuchi, T. , Aikawa, T. and Kanzaki, N. 2012. Simple and quick methods for nematode DNA preparation. Applied Entomology and Zoology 47:291–294, doi: 10.1007/s13355-012-0115-9.

[ref040] Wöhr, M. , Greeff, J. M. , Kanzaki, N. , Ye, W. and Giblin-Davis, R. M. 2014. Molecular and morphological observations of *Parasitodiplogaster sycophilon* Poinar 1979 (Nematoda: Diplogastrina) associated with *Ficus burkei* in Africa. Nematology 16:453–462, doi: 10.1163/15685411-00002777.

[ref041] Ye, W. , Giblin-Davis, R. M. , Braasch, H. , Morris, K. and Thomas, W. K. 2007. Phylogenetic relationships among *Bursaphelenchus* species (Nematoda: Parasitaphelenchidae) inferred from nuclear ribosomal and mitochondrial DNA sequence data. Molecular Phylogenetics and Evolution 43:1185–1197, doi: 10.1016/j.ympev.2007.02.006. 17433722

